# EHMTI-0136. Medical cannabis in the treatment of treatment-resistant, chronic cluster headache. A retrospective report

**DOI:** 10.1186/1129-2377-15-S1-C46

**Published:** 2014-09-18

**Authors:** A Mosek, M Dano, Y Fainmesser, Z Hybloom

**Affiliations:** 1 The Headache Clinic Dep. of Neurology, Tel Aviv Sourasky Medical Center, Tel-Aviv, Israel

## Background

Treatment-resistant chronic cluster headache is a debilitating disease. Since 2011 we were able to offer cannabis and we hereby report our experience using cannabis in this condition.

## Methods

A retrospective medical files analysis. The Institutional Review Board waived the need for informed consent.

## Results

Out of 120 patients seen with cluster-headache since 2011, 31 (26%) were diagnosed with chronic cluster-headache. Eighteen of them (78% men, average age 42.7 ±10 years), were resistant to conventional treatments for cluster-headache and initiated cannabis treatment. The average duration of the chronic pain was 8.5 ± 6 years (range 2-28 years). Twelve of the subjects had 1-6, daily or near-daily, attacks. The rest had 1-4 weekly attacks. Background daily headache accompanied 7 of the patients.

Smoking was the preferred modality of cannabis usage (average 1 gram/daily, range 0.7-1.7 grams/daily). In an average follow-up of 1.8 ± 0.8 years (2 months-3 years), cannabis usage resulted in >50% decrease of the headache severity in 15 (83%) patients, and in 90-100% severity decrease in 11 (61%) subjects. Nine (50%) patients reported 80-100% decrease in the headache frequency while the rest had no change in their attack frequency. Eleven patients (61%) reported high satisfaction from cannabis usage, with significant reduction of medications; sleep and quality of life improvement. Somnolence and dizziness limited cannabis usage in 2 subjects. Treatment success was unrelated to age, gender, duration of the headache or the chronic state.

## Conclusions

Cannabis was found an effective treatment for resistant chronic cluster-headache, with minimal adverse-events.

No conflict of interest.

